# Reconstruction of dental roots for implant planning purposes: a feasibility study

**DOI:** 10.1007/s11548-022-02716-x

**Published:** 2022-07-29

**Authors:** Leonard Simon Brandenburg, Lukas Berger, Steffen Jochen Schwarz, Hans Meine, Julia Vera Weingart, David Steybe, Benedikt Christopher Spies, Felix Burkhardt, Stefan Schlager, Marc Christian Metzger

**Affiliations:** 1 Department of Oral and Maxillofacial Surgery, Faculty of Medicine, Clinic, Medical Center –University of Freiburg, Hugstetterstr. 55, 79106 Freiburg, Germany; 2 Faculty of Medicine, University of Freiburg, Hugstetterstr. 55, 79106 Freiburg, Germany; 3 Fraunhofer Institute for Digital Medicine MEVIS, Am Fallturm 1, 28359 Bremen, Germany; 4 Department of Prosthodontics, Faculty of Medicine, Clinic, Medical Center –University of Freiburg, Hugstetterstr. 55, 79106 Freiburg, Germany; 5 Department of Oral and Maxillofacial Surgery, Albert-Ludwigs University Freiburg, Hugstetterstr. 55, 79106 Freiburg, Germany

**Keywords:** Implant planning, Statistical shape model, Anatomical reconstruction, Virtual planning

## Abstract

**Purpose:**

Modern virtual implant planning is a time-consuming procedure, requiring a careful assessment of prosthetic and anatomical factors within a three-dimensional dataset. In order to facilitate the planning process and provide additional information, this study examines a statistical shape model (SSM) to compute the course of dental roots based on a surface scan.

**Material and methods:**

Plaster models of orthognathic patients were scanned and superimposed with three-dimensional data of a cone-beam computer tomography (CBCT). Based on the open-source software “R”, including the packages Morpho, mesheR, Rvcg and RvtkStatismo, an SSM was generated to estimate the tooth axes. The accuracy of the calculated tooth axes was determined using a leave-one-out cross-validation. The deviation of tooth axis prediction in terms of angle or horizontal shift is described with mean and standard deviation. The planning dataset of an implant surgery patient was additionally analyzed using the SSM.

**Results:**

71 datasets were included in this study. The mean angle between the estimated tooth-axis and the actual tooth-axis was 7.5 ± 4.3° in the upper jaw and 6.7 ± 3.8° in the lower jaw. The horizontal deviation between the tooth axis and estimated axis was 1.3 ± 0.8 mm close to the cementoenamel junction, and 0.7 ± 0.5 mm in the apical third of the root. Results for models with one missing tooth did not differ significantly. In the clinical dataset, the SSM could give a reasonable aid for implant positioning.

**Conclusions:**

With the presented SSM, the approximate course of dental roots can be predicted based on a surface scan. There was no difference in predicting the tooth axis of existent or missing teeth. In clinical context, the estimation of tooth axes of missing teeth could serve as a reference for implant positioning. However, a higher number of training data must be achieved to obtain increasing accuracy.

**Supplementary Information:**

The online version contains supplementary material available at 10.1007/s11548-022-02716-x.

## Introduction

Positioning of oral implants is a challenging task and depends on a variety of anatomical and prosthodontic factors. In this context, the bone supply, soft tissue conditions, and the prospective position of the prosthetic restoration must be addressed in order to find the best possible spatial alignment for dental implants [1–5].

Computer-assisted planning and guided surgery alleviates the planning process and enables to transfer the digitally designed implant position into the oral cavity [6]. In order to visualize the osseous structures in 3D and to display adjacent anatomical structures of the implant site, a cone-beam computer tomography (CBCT) is routinely used for planning dental implant surgery. Current implant planning software solutions depict the patient’s anatomy and the planned restoration in one three-dimensional (3D) dataset and thus provide valuable anatomical and prosthodontic information for implant positioning [7]. The usefulness of this approach, which is associated with an increasing implant survival ratio, has been proven previously [8].

In the last decades, sophisticated software has been used increasingly in medicine to collect, compute and display patient specific data [9–12]. Statistical shape modeling (SSM) is one of the many developments in the field of medical computational networking and can be used for automated processing of medical image data. SSMs capture a high number of individual shapes of an anatomical structure and can be used to find similar shapes within a data set. SSMs model the average shape of an object and give a scope within which the shape is allowed to vary. Thus, in addition to mere segmentation of anatomical structures, a plausible shape of missing or damaged structures can be computed using SSMs [13].

The clinical use of SSMs for three-dimensional reconstruction of missing and/or fractured parts of the skull, orbits, and midface has been demonstrated previously [14–16]. Since each tooth has characteristic crown and root features, [17] the position and course of tooth roots could be estimated on the basis of the crown morphology using an SSM. The spatial covariance of dental crowns and roots could be captured in an SSM and enable the estimation of dental root morphology based on a surface scan of the tooth crowns. Recently, scientific articles have been published showing the possibility of reconstructing tooth crown morphology [18] and the mucogingival borderline [19] in clinical datasets using SSM.

In the context of implant planning, statistical shape modeling might predict the tooth axis of missing teeth, based on the tooth crown morphology of remaining teeth. This could serve as a reasonable orientation aid. Manual alignment of dental implants could be facilitated in presence of this anatomical reference.

Aim of this study was to assess if dental root anatomy of missing teeth can be predicted based on a surface scan using an SSM.

## Materials and methods

A retrospective review of electronic patient charts was conducted to acquire the datasets. Patients who were treated at the Department of Oral and Maxillofacial Surgery of the Medical Center of the University of Freiburg, Germany and received a dental plaster cast model and a corresponding CBCT-scan in patient history were further evaluated to be included in the study (see below). Additionally, one clinical implant surgery case was retrospectively analyzed with the SSM. The study was reviewed and conducted in accordance with the ethical standards of our institution. Ethical approval (No. 20–1342) for the implementation of this study was obtained from the Ethics Committee of the University of Freiburg, Germany. The manuscript was written according to the guidelines of the STROBE checklist for uniform and comprehensible documentation of observational studies [20].

### Inclusion and exclusion criteria

#### Data for generating and evaluating the SSM

All orthognathic cases of the Clinic for Oral and Maxillofacial Surgery of the […] between 2015 and 2019 were reviewed. If CBCT and plaster cast models of sufficient quality were available, patients were considered for implementation in the SSM. Type or severity of the malocclusion was no criterion for study inclusion. The following criteria had to be met by all individuals included in this study:

1. Minimum age of 18 years at the time of study implementation
2. Availability of CBCT scans depicting the entire maxilla and mandible, sufficient for performing a 3D segmentation
3. Availability of digitized plaster models created at the same time of the CBCT scan, which show the dental crowns without any artifacts
4. Harmoniously formed dental arches in the maxilla and mandible, with fully erupted teeth 17–27 and 37–47 (FDI-scheme)
5. No teeth replaced by dentures or implants

The absence of an informed consent, inadequate image quality such as motion artifacts, wrong positioning of the patient in the CBCT device or incomplete depiction of the jaws led to exclusion. Patients with missing teeth (except wisdom teeth), misalignment of single teeth, incomplete orthodontic treatment or alterations in tooth alignment between the time of the CBCT scan and the fabrication of plaster cast models were equally excluded.

#### Clinical cases

An implant surgery case of the Department of Oral and Maxillofacial Surgery of the Medical Center of the University of Freiburg, Germany was analyzed using the SSM to assess the potential clinical usefulness of the proposed workflow. Plaster casts of the preoperative stage, a corresponding CBCT scan and the virtual planning data had to be available for study inclusion. The implant planning was performed using CoDiagnostix Implant software (Dental Wings Inc., Montreal, Canada). A surgical guide was manufactured by 3D-printing (Eden 260 V, Stratasys, Eden Prairie, USA) to perform surgery.

### Image acquisition

The indication for CBCT-imaging was defined following the local standards and was given by the need for preoperative planning of orthognathic or dental implant surgery. The CBCT scans were performed using the 3D Accuitomo 170 CBCT-scanner (Morita Corporation, Osaka, Japan), with a slice thickness of 0.25 mm and a FOV of 17 × 12 cm. Datasets were exported as DICOM-files (Digital Imaging and Communications in Medicine) from the local picture archiving and communication system (Agfa HealthCare IMPAX EE R20 XVII SU4, Mortsel, Belgium).

### Production of plaster casts and digitization

The plaster casts were produced using Alginoplast® (Kulzer Mitsui Chemicals Group, Hanau, Germany) and pico-crema soft® type 3 DIN EN ISO 6873 (Picodent, Wipperfürth, Germany). Digitization was performed using the E3 3-shape optical scanner (3shape, Copenhagen, Denmark) and exported as standard tessellation language (STL) files.

### Processing of datasets

#### Superimposition of CBCT and surface scan

**Fig. 1:**
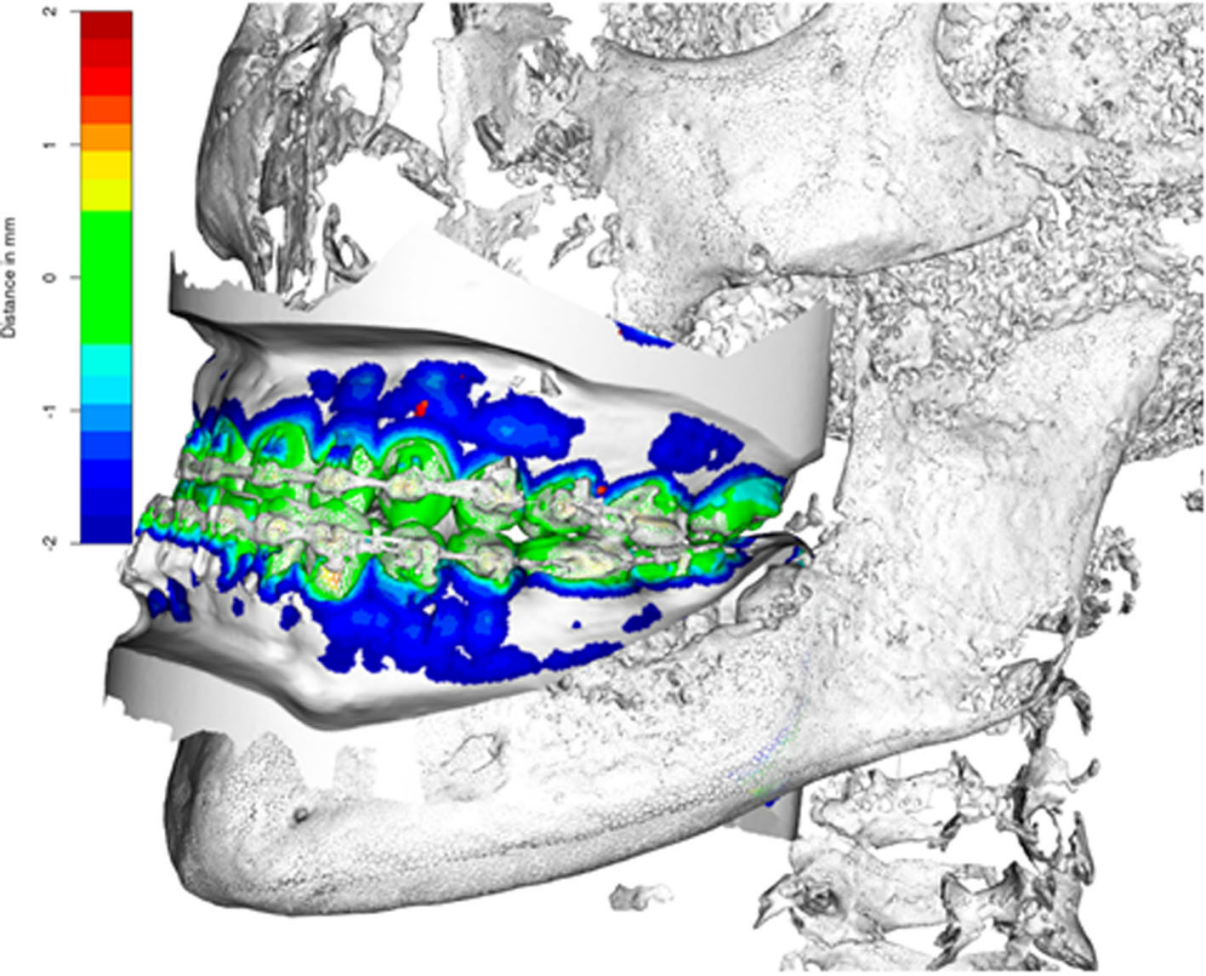
Superimposition of plaster cast and segmented CBCT scan. The deviation of the teeth surfaces after registration was throughout below 0.05 mm

The CBCT scans were mainly afflicted by artifacts due to vestibular attached fixed orthodontic appliances, leading to unsatisfying depiction of the tooth crown. Thus, to enable the accurate depiction of tooth crown morphology and dental root shape in one dataset, the CBCT had to be fused with the surface scan. The DICOM dataset was imported into 3D Slicer [21], and a segmentation of all bony structures and teeth was performed using the threshold segmentation tool. To fuse the segmented model of the CBCT-scan with the digitized plaster cast, five corresponding landmarks on both datasets were set and an iterative closest point (ICP) procedure was performed [22]. For ICP, only the lingual surfaces of the teeth were used, as they were not afflicted by metal artifacts due to the vestibular attached orthodontic appliances. The ICP iteratively reduces the distance between the teeth surfaces, resulting in a superimposition with a maximum distance below 0.5 mm (s. Fig. 1). As a result, a combined model containing detailed information of the tooth crown and the root was created.

#### Placement of anatomical landmarks

**Fig. 2:**
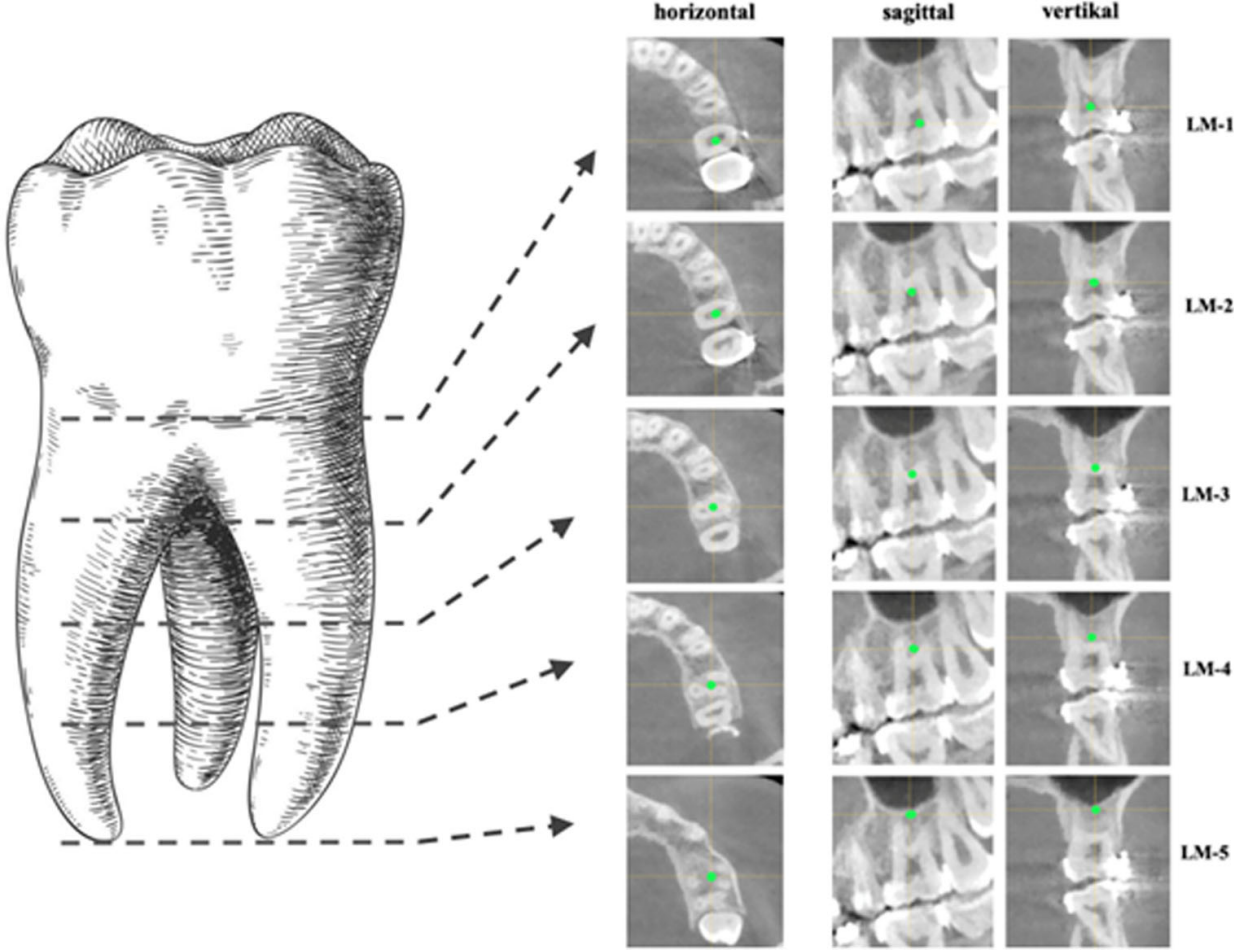
Procedure for placing landmarks along the roots of multi-rooted teeth. The first landmark was set at the level of the CEJL. Three further landmarks were placed with 2 mm distance along the apical direction. A fifth landmark was set at the mid-point of the apical end of all roots

**Fig. 3:**
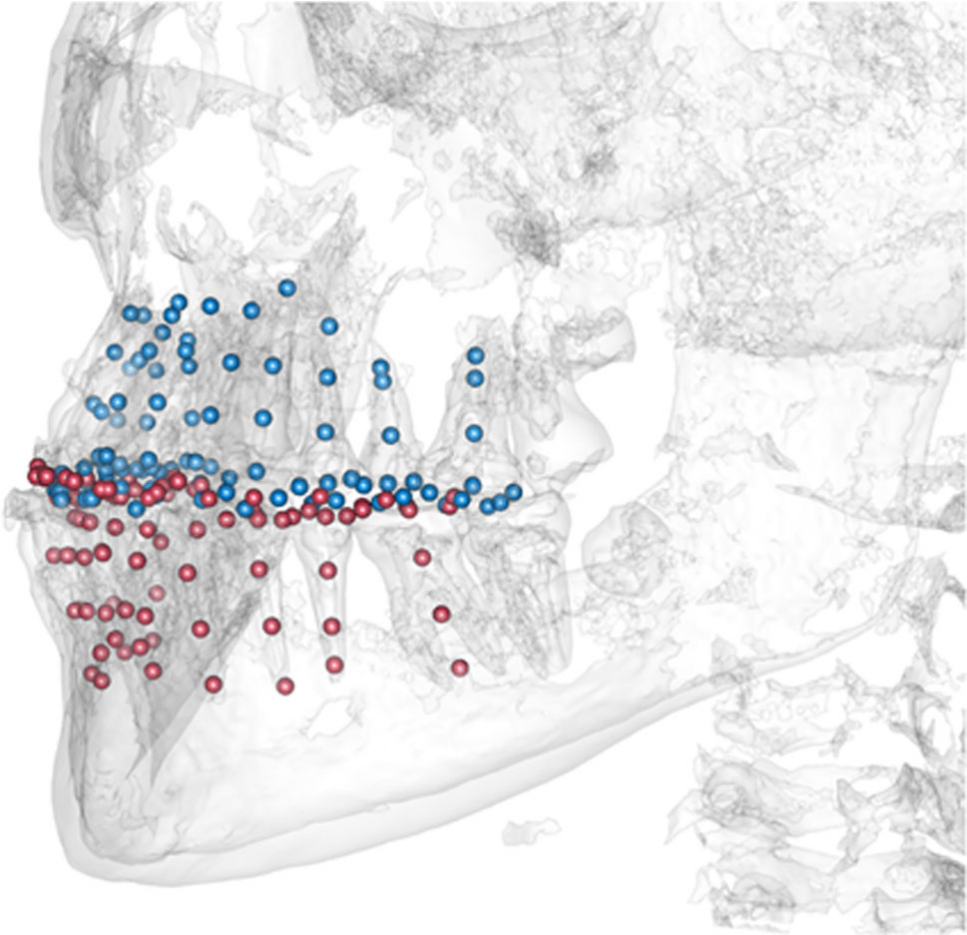
By augmentation of the CBCT-scan with the corresponding plaster cast model, all structures of the teeth could be captured with landmarks. Thus, every individual received 270 landmarks to encode the morphology of the tooth crowns and teeth

Anatomical landmarks were used to parametrize the complex morphology of the dental crowns and roots and to generate a landmark-based SSM of dental tooth shape. Dental cusps, incisal edges and central fissures were reliably detectable in the surface scan and were used to place uniform and reproducible landmarks at the tooth crown. A detailed description of the used landmarks is provided in the attached landmark protocol. The landmark-based description of the dental root anatomy was performed differently, because no unambiguously detectable anatomical landmarks are present along dental roots: five landmarks were defined for each dental root. A first landmark was placed in the center of the root canal at the level of the cemento-enamel-junction-line (CEJL). Three further landmarks, each two millimeters apart along the tooth root in the apical direction, were positioned in the center of the root canal. A fifth landmark was placed at the apical end of the root. Multi-rooted teeth were treated as if they had one large root when placing the landmarks. If several roots of the same tooth were cut in the horizontal section, the landmark was placed in their center (see Fig. 2). Eventually for each dataset, a landmark-based model was created containing information about the dental crown and root shape. Figure 3 depicts the landmarks of one individual within the CBCT dataset.

#### Calculation of tooth axes

In order to determine the dental root course, an idealized dental root axis was calculated for each tooth based on the landmarks LM-1–LM-4 using an OLS-regression line. This procedure allows minor errors in the subjective positioning of the root-landmarks in the CBCT-scan to be compensated for, since multiple landmarks represented the axis. Even if one landmark was incorrectly positioned, that effect would be mitigated by the remaining three landmarks.

### Generation of the SSM

The SSM generation and all analyses were performed using open-source software packages for the statistical/mathematical platform “R” (v. 4.0), specifically the packages Morpho, mesheR, Rvcg and RvtkStatismo [23–28]. All datasets were aligned in a common coordinate system by removing translation and rotation within space applying a Procrustes Registration [29]. The scaling of the datasets was not altered by this process, because it was assumed that the shape of a large jaw does not equal to a magnification of a smaller one and vice versa. Finally, the registered surface meshes were rigidly aligned, and the SSM was generated from a subsequent principal component analysis [30] (for details about the generation of SSMs see Cootes and Taylor 2004 [13]).

### Application and evaluation of the SSM

The SSM captures the shape variability present in the training data. Hereby, the mentioned landmarks encode the shape relationship between the surface scan and the information about the dental root given by the CBCT-scan.

Based on that information, the axis and position of the roots of the teeth can be estimated, regarding the information given by a surface scan. In particular, this should also be possible if a tooth is missing.

**Fig. 4:**
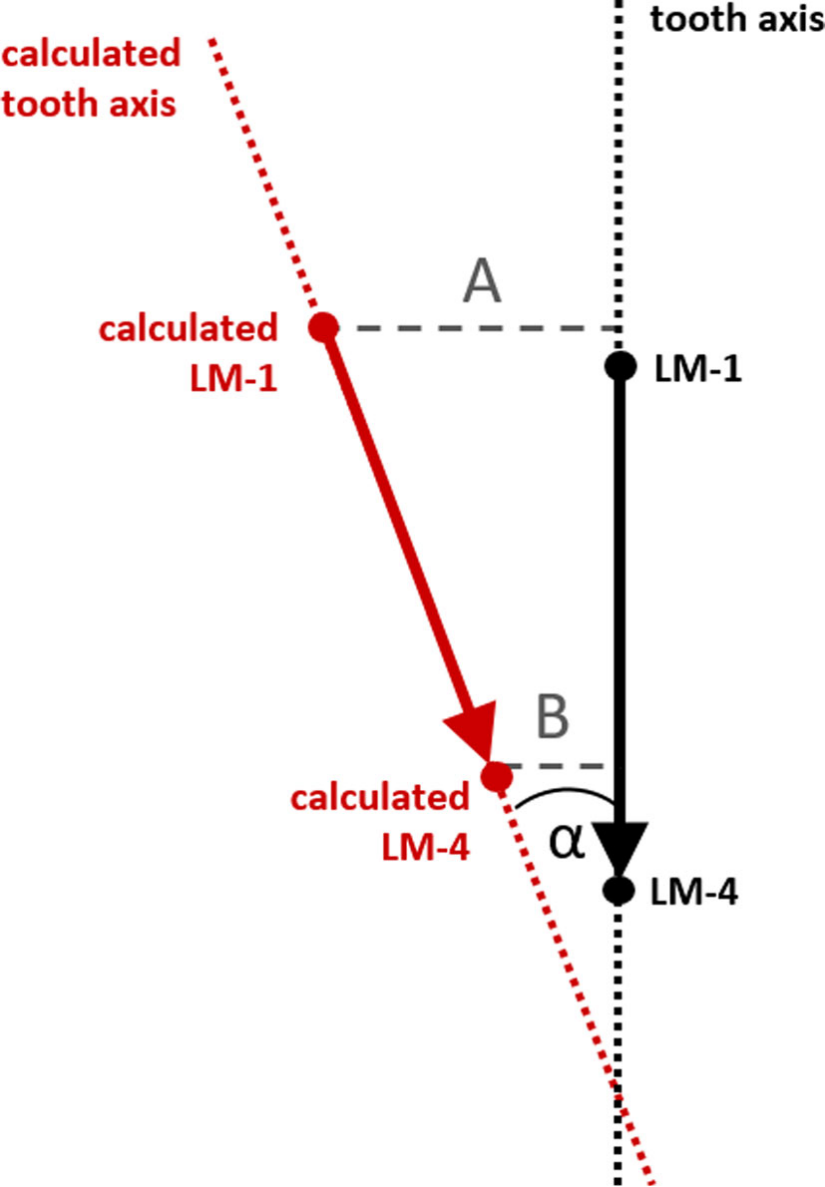
Measurements performed for evaluation of the calculated tooth axis (red) in comparison with the actual tooth axis (black). One section each at the level of the CEJL and the apical landmark LM-4 were measured, as well as the angular deviation between the two axes

For assessing the accuracy of our proposed approach, a leave-one-out cross-validation (LOOCV) was performed. LOOCV excludes one dataset at a time and generates an alternative SSM from the remaining datasets to avoid self-inference of the use case and the SSM. The excluded data set is then reconstructed using that SSM. The landmarks on the tooth crowns of the excluded model are taken as given, and the missing position and axis of the tooth root are to be determined. The calculated position and axis of the tooth root is then compared with the position and axis depicted by the CBCT-scan. To evaluate the reconstruction accuracy, the following parameters were determined (see Fig. 4):

- Angle alpha: angular deviation between the calculated and the real tooth axis
- Distance A: shortest distance between the calculated LM-1 and the actual tooth axis
- Distance B: shortest distance between the calculated LM-4 and the actual tooth axis

#### Evaluation of axis prediction in fully dentate models using LOOCV

In the first round, the excluded dataset was annotated with all crown landmarks for subsequent axis reconstruction. The prediction of the dental root axes was based on the complete set of 14 teeth per jaw. Evaluation of the reconstruction results was performed for all 14 teeth per jaw simultaneously according to the above-mentioned evaluation measures (Fig. 4). Results are given for the upper and lower jaw separately and for all teeth summarized as well as for each region individually. Each value was indicated as mean value with the according standard deviation.

#### Evaluation of axis prediction for missing teeth using LOOCV

In the second round, the performance of the SSM was tested after removing the landmarks of one tooth from the excluded dataset. In this case, the estimation of the respective root axis and its position is based only on the landmarks of the neighboring teeth. This was tested for all teeth within the excluded dataset individually (17–27 in the upper jaw, 37–47 in the lower jaw). The results are given for the upper and lower jaw separately, for all teeth summarized as well as for each region individually. The measurements for each evaluation criterion are summarized via mean value and the corresponding standard deviation.

To investigate differences between the groups (fully toothed vs. missing tooth) and among the different tooth regions, a paired t-test was applied. We corrected for alpha-inflation using the Bonferroni–Holm method [31].

### Clinical case

The dataset of a patient with a tooth gap from 12–11 was analyzed using the SSM, to test the potentials for clinical use (Figs. 7, 8, 9, 10). The axes which were reconstructed by the SSM were compared to the actual axis of existent teeth and to the planned implantation axis in the region of missing teeth.

## Results

A total of 230 patients received a CBCT-scan and a corresponding plaster cast model of the upper and lower jaw in the mentioned collection period. 159 cases were excluded because they did not meet imaging requirements (49 because of inadequate CBCT-scans, 42 because of inadequate plaster casts, 18 because of alterations in tooth alignment between the CBCT-scan and the plaster cast model and 50 due to missing premolars). Finally, 71 datasets underwent further processing and were included in the generation of the SSM. The mean age of all patients was 23.3 ± 6.1 with a median of 22 years. 34 women and 37 men were included. 130 and 140 landmarks, respectively, were set in each of the surface scans and the CBCT-scans for creating the landmark-based SSM of dental crowns and roots, leading to a total number of 19,170 landmarks placed for SSM-generation.

### Accuracy of axis prediction in fully dentate models using LOOCV

The accuracy of the SSM-based reconstruction in fully dentate models summarized for all teeth was as follows:

- In the upper jaw: mean angle alpha = 7.5 ± 4.3 degrees, mean distance A = 1.3 ± 0.8 mm, mean distance B = 0.7 ± 0.5 mm.
- In the lower jaw: mean angle alpha = 6.7 ± 3.8 degrees, mean distance A = 1.2 ± 0.8 mm, mean distance B = 0.7 ± 0.6 mm.

The accuracy of the SSM-based reconstructions in fully dentate models in dependence of the reconstructed region is shown in Table 1.

**Table 1:** Reconstruction accuracy of tooth axes specified per tooth for fully toothed dental arches (left columns) and for plaster casts with one missing tooth (right columns)

Tooth-axis	Fully toothed dental arches				Models with one tooth missing			
	Mean angle alpha	SD angle alpha	Mean distance A	SD distance A	Mean angle alpha	SD angle alpha	Mean distance A	SD distance A
11	6.2	4.0	0.7	0.5	6.3	4.1	0.7	0.5
12	7.7	4.8	0.8	0.4	7.8	4.8	0.8	0.5
13	6.0	3.5	0.7	0.5	6.2	3.5	0.8	0.5
14	7.5	3.5	0.7	0.5	7.7	3.7	0.7	0.4
15	7.3	3.8	0.7	0.5	7.4	3.9	0.7	0.5
16	7.7	3.6	0.8	0.6	8.2	3.8	0.9	0.7
17	9.3	4.3	1.0	0.9	9.6	4.4	1.4	0.8
21	7.0	4.6	0.7	0.4	7.1	4.3	0.7	0.5
22	6.7	4.3	0.8	0.4	6.8	4.2	0.8	0.5
23	7.0	4.6	0.6	0.4	7.3	5.0	0.7	0.4
24	7.5	4.1	0.7	0.4	7.6	4.3	0.7	0.4
25	7.3	4.1	0.6	0.4	7.5	4.1	0.7	0.4
26	8.9	4.4	0.6	0.4	9.2	4.5	0.8	0.4
27	9.2	5.2	0.7	0.5	10.1	5.3	1.2	0.6
31	5.4	3.4	0.6	0.4	5.7	3.6	0.6	0.4
32	5.9	3.0	0.7	0.4	6.1	3.2	0.8	0.4
33	6.1	3.7	0.7	0.4	6.4	3.8	0.7	0.4
34	7.1	4.0	0.6	0.3	7.2	4.0	0.6	0.4
35	7.1	3.7	0.7	0.4	7.4	3.9	0.7	0.4
36	7.8	4.0	0.7	0.6	9.2	4.9	0.9	0.7
37	8.6	4.5	0.9	0.9	9.2	5.2	1.2	0.8
41	5.3	3.2	0.6	0.4	5.5	3.3	0.6	0.4
42	5.6	3.2	0.6	0.3	5.8	3.2	0.7	0.4
43	5.8	2.9	0.7	0.3	6.3	3.0	0.7	0.3
44	6.4	3.3	0.6	0.3	6.5	3.4	0.6	0.3
45	6.5	3.6	0.6	0.4	6.8	3.8	0.7	0.4
46	7.6	4.3	0.7	0.6	8.6	4.6	0.8	0.7
47	7.9	4.2	1.0	1.1	9.5	5.1	1.3	1.0

**Fig. 5:**
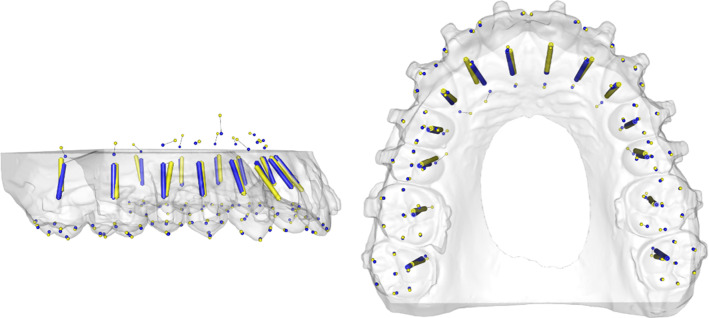
Example for the reconstruction of a fully toothed upper arch. Predicted axes (blue) and actual axes (yellow) are shown in one dataset

**Fig. 6:**
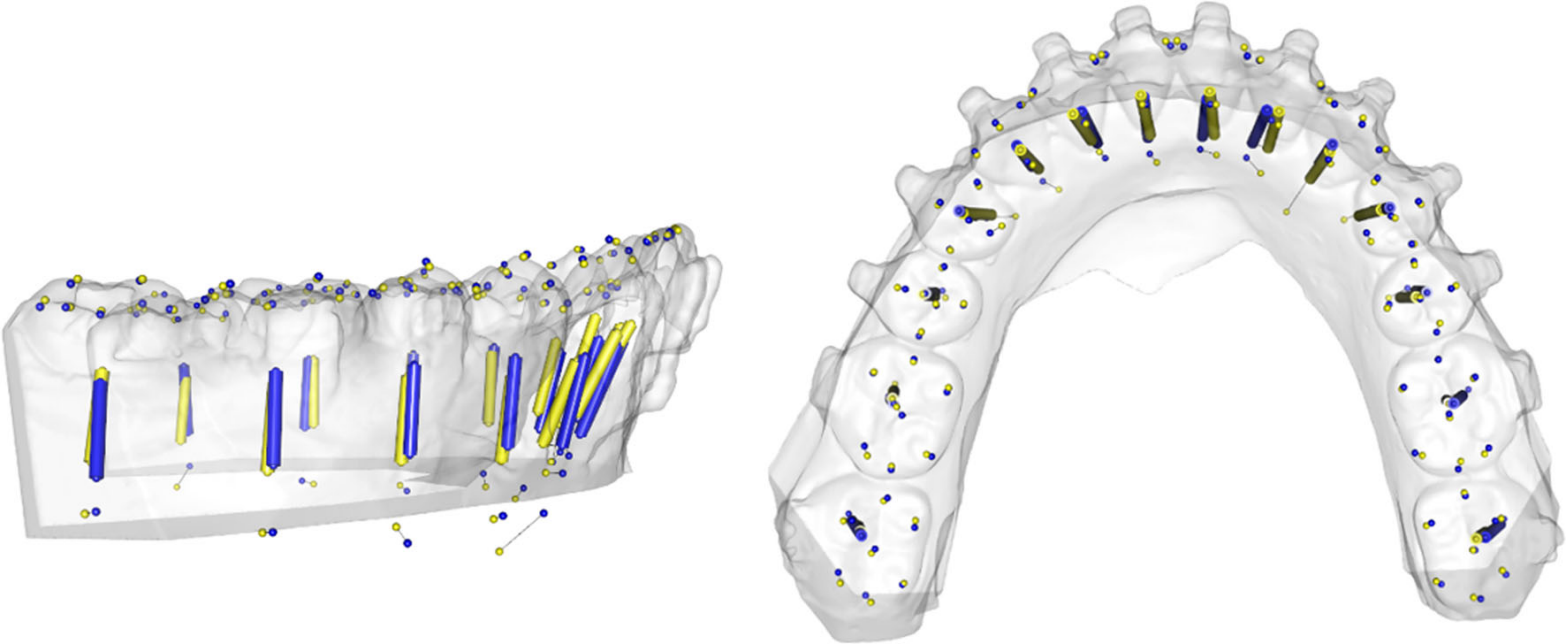
Example for the reconstruction of a fully toothed lower arch. Predicted axes (blue) and actual axes (yellow) are shown in one dataset

One of the reconstructed datasets is depicted in Figs. 5, 6 exemplarily. (Fig. 5a and b shows the reconstructed and real tooth axes of the upper jaw. Figure 6a and b shows the reconstructed and real tooth axes of the lower jaw.)

### Accuracy of axis prediction for missing teeth using LOOCV

Incomplete plaster casts were used to reconstruct the tooth axis of a missing tooth, leading to 14 reconstruction scenarios per jaw. The reconstruction accuracy is specified per tooth in Table 1 and can be described in summary for all different regions as follows:

- In the upper jaw: mean angle alpha = 7.8 ± 4.4 degrees, mean distance A = 1.3 ± 0.8 mm, mean distance B = 0.8 ± 0.6 mm.
- In the lower jaw: mean angle alpha = 7.2 ± 4.2 degrees, mean distance A = 1.3 ± 0.8 mm, mean distance B = 0.8 ± 0.6 mm.

The accuracy of the SSM-based reconstructions in models with one missing tooth in dependence of the reconstructed region is shown in Table 1. There were no significant differences between the group with fully toothed arches and the group with one missing tooth regarding the reconstruction accuracy (p > 0.05).

### Clinical case

**Fig. 7:**
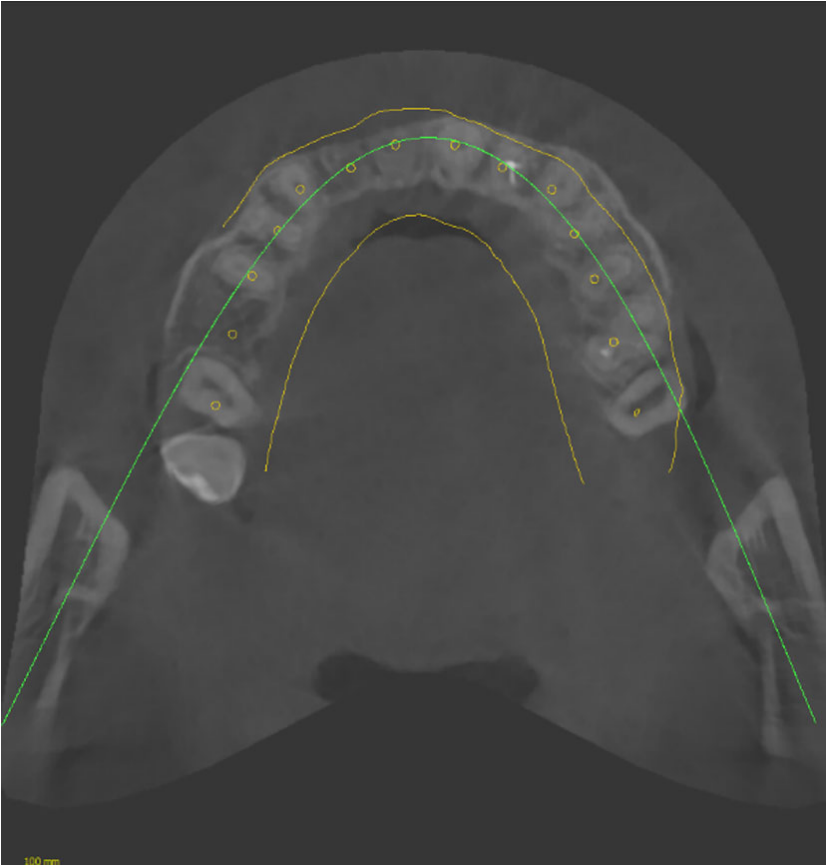
Using CoDiagnostix, a superimposition of the CBCT-scan with the axes reconstructed by the SSM and a dental wax up (both in yellow) was performed. The axes calculated by the SSM were depicted as yellow cylinders. Therefore, in this horizontal cross section, they appear as a dot. The yellow dots mostly match with the hypodense area of the root canals of the teeth (if existent)

**Fig. 8:**
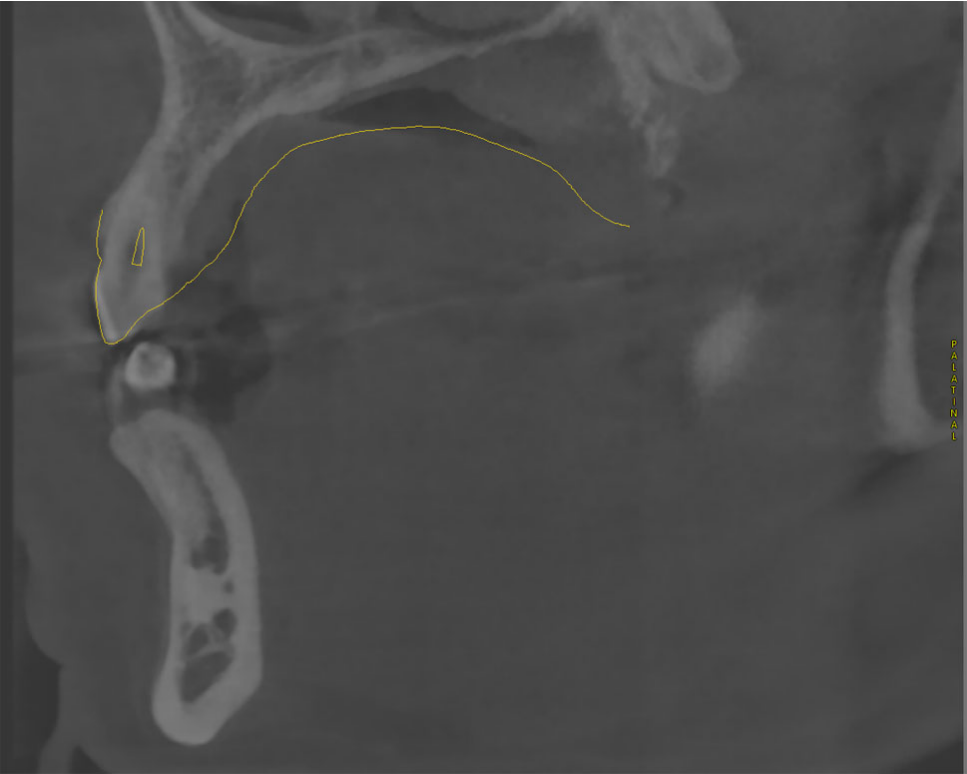
Paramedian cut of the CBCT-scan superimposed with a dental wax-up and the SSM-based tooth axis reconstruction. The predicted tooth axis of tooth 23 (yellow) is located at a similar position as the actual root canal of the tooth 23. The comparison of calculated tooth axis with dental roots of existent teeth enables to presume the validity of the SSM-based prediction of missing roots

**Fig. 9:**
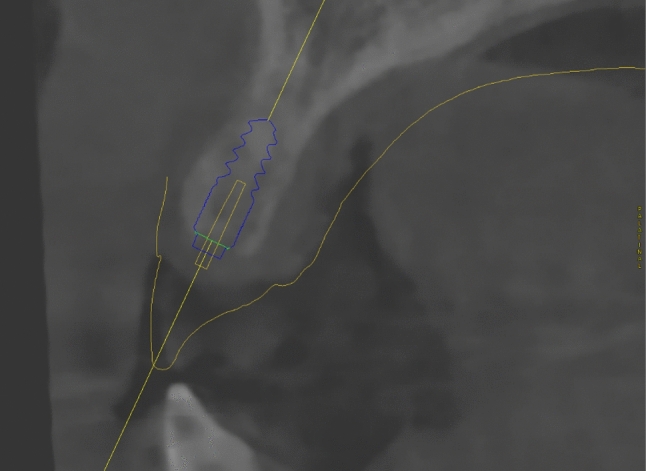
Paramedian cut of the CBCT-scan superimposed with a dental wax-up and the SSM-based tooth axis reconstruction (both in yellow). The SSM-based prediction of the tooth axis of tooth 12 appears to be close to parallel to the planned implantation axis

**Fig. 10:**
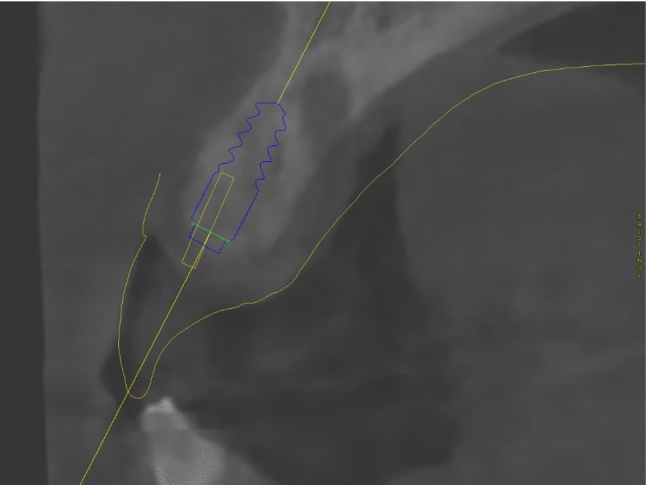
Paramedian cut of the CBCT-scan superimposed with a dental wax-up and the SSM-based tooth axis reconstruction (both in yellow). The SSM-based prediction of the tooth axis of tooth 11 deviates from the planned implantation axis in oro-vestibular direction. Due to vestibular bone loss, the implantation axis was adjusted to guarantee sufficient bone thickness in all dimensions

A patient with a tooth gap from 12–11 was included for the clinical assessment of the SSM. The tooth axes of present and missing teeth of the upper jaw were calculated using the SSM and projected into the digital planning dataset. The superimposed data shows that the SSM calculates an approximate replica of the actual tooth axis of present teeth (Figs. 7, 8). The axis of the implant in region 12 was close to identical with the calculated axis for tooth 12 (Fig. 9). The SSM-based prediction of tooth axis 11 differed from the planned implantation axis in oro-vestibular direction (Fig. 10).

## Discussion

### Main findings

In this study, the estimation of dental root axes based on surface scans using an SSM was demonstrated. The morphology of teeth was encoded within the SSM by anatomical landmarks placed along the crowns and roots of all teeth of the study group. Thus, the SSM could be deployed to estimate missing landmarks of dental roots according to the shape variability of the underlying training data. Regardless of the location of the respective tooth in the maxilla or mandible, the average estimation error was below 8° angular and 1.4 mm translational deviation. No statistically significant differences could be found regarding the tooth axis prediction error in different tooth regions. Even if it was to be assumed that the calculation of an average axis of a multi-rooted tooth could compensate for errors due to special root features, the accuracy for estimation of tooth axis in single-rooted teeth was comparably accurate.

Surprisingly, the virtual removal of one tooth in the landmark dataset did hardly affect the reconstruction results. Thus, the presented method for estimating the dental root axes appears to be robust even if single tooth gaps are present. With the proposed workflow, the original axis of missing teeth can therefore be predicted.

Moreover, the reconstruction of tooth axes using a SSM could be implemented in the digital planning process of a clinical case where it could serve as a valuable orientation aid for virtual implant placement.

### Comparison to other workflows

For depicting the course of dental roots, CBCT is a well-validated imaging technique enabling accurate three-dimensional assessment.

In the current literature, only few publications concern the estimation of dental root axes based on surface scans:

For orthodontic follow-up examinations, another workflow was presented to depict tooth axes based on surface scans. Initial CBCT scans were superimposed with their corresponding surface scan, and a 3D-model of each tooth was created. These individual models can be fused with surface scans of other treatment stages, showing the hidden course of the approximate tooth root inside the hard tissues of the jaws [32]. The advantage of this approach is that this workflow considers the actual shape of the dental roots instead of computing it with the accompanied inaccuracies. However, this workflow requires at least one high-resolution CBCT scan of the jaws to generate individual tooth models and cannot forgo radiation completely.

While there is a patent which describes the use of 3D shape modeling to predict a 3D dental arch, including roots, from 2D photographs, it does not contain any ground truth or error assessment and seems to be aimed to generate computer graphics effects for video games or movies [33].

Ortho Insight 3D software (Motion View LLC, Chattanooga, Tennessee, USA) tries to predict the shape of tooth roots using standard tooth models [34, 35]. Studies which investigated the accuracy of this approach report deviations of 9.2—22.5 degrees [34], respectively, 7.95 – 15.28 degrees [35] between the actual and the estimated root axis in mean. Despite having comparably poor accuracy, this tool is already implemented in a commercial software for quick calculation of dental root course based on a surface scan. As we already achieved significantly higher accuracy for tooth axis prediction in this feasibility study (6.0 – 8.6 degrees in mean), the presented method appears to be promising for future clinical application. Ongoing research focuses on the implementation of a larger number of training data, to achieve higher accuracy.

### Limitations of the study

As a feasibility study, this study does not claim to present a technically fully developed and clinically usable workflow. To investigate the initial feasibility of tooth axis reconstruction using a SSM, several limitations had to be accepted:

#### Study group

To avoid a patient consultation for study purposes and forgo the associated additional radiation exposure, the SSM was created with retrospectively available data. Unfortunately, the digitization of our outpatient clinic was not yet completed at the time of data collection, so that scans of conventionally fabricated plaster casts had to be used. The use of intraoral scans would have forgone cumbersome and erroneous digitization of plaster casts. Therefore, we recommend the use of intraoral scanners for future studies in the field. Moreover, in the Department of Oral and Maxillofacial Surgery of the Medical Center of the University of Freiburg, Germany only orthognathic patients received a simultaneous CBCT scan and correlating plaster casts, so data acquisition was limited to orthognathic patients. All of them received orthodontic treatment in the preoperative stage. Orthodontic appliances were covered with boxing wax prior to taking dental impressions. Thus, the plaster models produced did not show the actual tooth shape in the vestibular area. Nevertheless, the SSM managed to calculate the dental root axes, even if the training data were afflicted by these artifacts. To guarantee this performance, the occlusal and oral surfaces of the teeth had to be depicted without any artifacts. Further work should be done to extend the training data and collect anatomical datasets of healthy individuals without any orthodontic appliances to ensure a reliable workflow. As orthodontic treatment may have an impact on the periodontal status and tooth morphology, a potential bias based on the selection of the study group has to be considered [36]. Nevertheless, to avoid additional radiation exposure of healthy individuals, this feasibility study was restricted to retrospectively available data. Thus, a limited number of cases could be included in the SSM. For more accurate reconstruction results, the underlying study group should be expanded with healthy individuals in future studies.

#### Application of anatomical landmarks

To enable a first assessment of tooth axis reconstruction using a SSM, the complex anatomy of human teeth was extrapolated by a set of landmarks. Even though a multitude of landmarks was set at each tooth, this approach cannot reconstruct a three-dimensional surface of dental roots. Especially in implant surgery, the detailed shape and course of dental roots can be crucial to avoid damage on adjacent teeth. An SSM containing the full shape information of dental crowns and roots would give more detailed information about the morphology of the teeth. The placement of the landmarks depends on the manual interaction of the operator. If set with poor accuracy, the landmarks could generate additional noise and introduce unwanted variability in the SSM.

#### Calculation of tooth axes

In order to represent each tooth and its corresponding tooth axis as a whole, multi-rooted roots were considered as one unit. On the one hand, this facilitates considerations regarding the biomechanical axis of the tooth, but, on the other hand, it is a simplification of the far more complex anatomy of multi-rooted teeth. To receive information about the location of each of the sprouts of multi-rooted teeth, a more sophisticated procedure is required to take the complex anatomy of all teeth into account. On the other hand, to use this workflow for implant axis prediction, the complex anatomy of multi-rooted teeth must eventually be simplified in terms of a single axis.

#### Generalizability

Due to the retrospective design of this study, the training data were restricted to a small group of patients, for which the required data were available. Even if the calculation of tooth axes was performed successfully using the presented workflow, it has to be assessed whether the generated SSM works reliably for other patients who are not diagnosed with orthognathic malformation. Therefore, the results of the clinical case investigated in this study do not proof that the presented workflow is generally applicable on all patients. Further work has to be done to collect and implement a high number of datasets from many different individuals to capture all morphological variations of oral anatomy and extend the SSM.

### Potentials for application in implant planning

A possible application for SSM-based root predictions could be dental implantology. Positioning of an implant is limited by anatomical structures in all three axes of space, leaving a certain leeway space for implant positioning:

The first axis (x-axis) describes the mesio-distal position of the implant. Adjacent teeth limit the leeway space in this axis [37, 38]. Undercutting the safety distances to neighboring teeth or implants can result in bone resorption and loss of blood supply, yielding to a reduced stability of the implant. [37]

The second axis (y-axis) is described by the oro-lingual width of the alveolar ridge. Proper positioning along this axis is crucial in order to get a good aesthetic outcome. [1, 2].

The drilling depth and the axial length of the implant concern the third axis of space (z-axis). Coronal the implant should be in one plane with the crestal bone level. On the apical end, safety distances to the inferior alveolar nerve, respectively, the maxillary sinus should be maintained [39].

To guarantee the safety distances in all three axes of space, the positioning of dental implants along the original tooth axis can be reasonable from a biomechanical and topographical point of view [40]. The proposed workflow using an SSM can reconstruct the course of dental roots of missing teeth, which could serve as a template for implant placement. Moreover, the surgeon can derive valuable information from anatomical tooth axis reconstruction. For example, the necessity for bone augmentation can be revealed.

However, the accuracy of the workflow has to be improved by including more training data to meet the standards of current implant surgery.

## Conclusion

In conclusion, it can be stated that the presented method supplies an automatic method for estimating the course of dental roots of missing teeth with an accuracy below 8 degrees angular deviation, respectively, 1.4 mm translational shift. The presented SSM computes teeth axes regarding only the morphology of remaining tooth crowns and could serve as a useful tool for automated and reasonable positioning of implants within a three-dimensional dataset. Current efforts focus on increasing the accuracy and enable clinical access of the proposed method. To the best of our knowledge, there are no publications reporting of a method for accurately estimate the tooth axis of missing teeth.

## Supplementary Information

Below is the link to the electronic supplementary material.

Supplementary file1 (DOCX 29 KB)
