# Supplementary material for: Reconstruction of dental roots for implant planning purposes: a feasibility study
Source: Int J Comput Assist Radiol Surg. 2022 Jul 29;17(10):1957–68. doi: 10.1007/s11548-022-02716-x (PMC9468133; doi:10.1007/s11548-022-02716-x)
Supplement: Supplementary file 1 — Supplementary file1 (DOCX 29 KB) [file 11548_2022_2716_MOESM1_ESM.docx]

**Landmark Protocol**

**Landmarks used for fusion of CBCT-dataset and surface scan**

**Landmarks CBCT:**

toothcrown_CBCT_lm-1: mb cusp maxilla 7 right

toothcrown_CBCT_lm-2: dental cusp maxilla 3 right

toothcrown_CBCT_lm-3: incisal point maxilla maxilla

toothcrown_CBCT_lm-4: dental cusp maxilla 3 left

toothcrown_CBCT_lm-5: mb cusp maxilla 7 left

toothcrown_CBCT_lm-6: mb cusp mandible 7 left

toothcrown_CBCT_lm-7: dental cusp mandible 3 left

toothcrown_CBCT_lm-8: incisal point mandible mandible

toothcrown_CBCT_lm-9: dental cusp mandible 3 right

toothcrown_CBCT_lm-10: mb cusp mandible 7 right

**Landmarks plaster cast:**

toothcrown_plaster_lm-1: mb cusp maxilla 7 right

toothcrown_plaster_lm-2: dental cusp maxilla 3 Right

toothcrown_plaster_lm-3: incisal point maxilla maxilla

toothcrown_plaster_lm-4: dental cusp maxilla 3 left

toothcrown_plaster_lm-5: mb cusp maxilla 7 left

toothcrown_plaster_lm-6: mb cusp mandible 7 left

toothcrown_plaster_lm-7: dental cusp mandible 3 left

toothcrown_plaster_lm-8: incisal point mandible mandible

toothcrown_plaster_lm-9: dental cusp mandible 3 right

toothcrown_plaster_lm-10: mb cusp mandible 7 right

**Landmarks set at tooth crowns on the plaster cast model**

**Maxilla**

arch_plaster_17_lm-1 distobuccal dental cusp 17

arch_plaster_17_lm-2 mesiobuccal dental cusp 17

arch_plaster_17_lm-3 distal edge 17

arch_plaster_17_lm-4 midpoint of central fissure 17

arch_plaster_17_lm-5 mesial edge 17

arch_plaster_17_lm-6 distolinduale dental cusp 17

arch_plaster_17_lm-7 mesiolingual dental cusp 17

arch_plaster_16_lm-1 distobuccal dental cusp 16

arch_plaster_16_lm-2 mesiobuccal dental cusp 16

arch_plaster_16_lm-3 distal edge 16

arch_plaster_16_lm-4 midpoint of central fissure 16

arch_plaster_16_lm-5 mesial edge 16

arch_plaster_16_lm-6 distolingual dental cusp 16

arch_plaster_16_lm-7 mesiolingual dental cusp 16

arch_plaster_15_lm-1 buccal dental cusp 15

arch_plaster_15_lm-2 distal edge 15

arch_plaster_15_lm-3 midpoint of central fissure 15

arch_plaster_15_lm-4 mesial edge 15

arch_plaster_15_lm-5 lingual dental cusp 15

arch_plaster_14_lm-1 buccal dental cusp 14

arch_plaster_14_lm-2 distal edge 14

arch_plaster_14_lm-3 midpoint of central fissure 14

arch_plaster_14_lm-4 mesial edge 14

arch_plaster_14_lm-5 lingual dental cusp 14

arch_plaster_13_lm-1 distal contact point 13

arch_plaster_13_lm-2 buccal dental cusp 13

arch_plaster_13_lm-3 mesial contact point 13

arch_plaster_13_lm-4 tuberculum lingual 13

arch_plaster_12_lm-1 distal incisal edge 12

arch_plaster_12_lm-2 mesial incisal edge 12

arch_plaster_12_lm-3 tuberculum lingual12

arch_plaster_11_lm-1 distal incisal edge 11

arch_plaster_11_lm-2 mesial incisal edge 11

arch_plaster_11_lm-3 tuberculum lingual 11

arch_plaster_21_lm-1 mesial incisal edge 21

arch_plaster_21_lm-2 distal incisal edge 21

arch_plaster_21_lm-3 tuberculum lingual 21

arch_plaster_22_lm-1 mesial incisal edge 22

arch_plaster_22_lm-2 distal incisal edge 22

arch_plaster_22_lm-3 tuberculum lingual 22

arch_plaster_23_lm-1 mesial contact point 23

arch_plaster_23_lm-2 buccal dental cusp 23

arch_plaster_23_lm-3 distal contact point 23

arch_plaster_23_lm-4 tuberculum lingual 23

arch_plaster_24_lm-1 buccal dental cusp 24

arch_plaster_24_lm-2 mesial edge 24

arch_plaster_24_lm-3 midpoint of central fissure 24

arch_plaster_24_lm-4 distal edge 24

arch_plaster_24_lm-5 lingual dental cusp 24

arch_plaster_25_lm-1 buccal dental cusp 25

arch_plaster_25_lm-2 mesial edge 25

arch_plaster_25_lm-3 midpoint of central fissure 25

arch_plaster_25_lm-4 distal edge 25

arch_plaster_25_lm-5 lingual dental cusp 25

arch_plaster_26_lm-1 mesiobuccal dental cusp 26

arch_plaster_26_lm-2 distobuccal dental cusp 26

arch_plaster_26_lm-3 mesial edge 26

arch_plaster_26_lm-4 midpoint of central fissure 26

arch_plaster_26_lm-5 distal edge 26

arch_plaster_26_lm-6 mesiolingual dental cusp 26

arch_plaster_26_lm-7 distolingual dental cusp 26

arch_plaster_27_lm-1 mesiobuccal dental cusp 27

arch_plaster_27_lm-2 distobuccal dental cusp 27

arch_plaster_27_lm-3 mesial edge 27

arch_plaster_27_lm-4 midpoint of central fissure 27

arch_plaster_27_lm-5 distal edge 27

arch_plaster_27_lm-6 mesiolingual dental cusp 27

arch_plaster_27_lm-7 distolingual dental cusp 27

**Mandible**

arch_plaster_37_lm-1 distobuccal dental cusp 37

arch_plaster_37_lm-2 mesiobuccal dental cusp 37

arch_plaster_37_lm-3 distal edge 37

arch_plaster_37_lm-4 midpoint of central fissure 37

arch_plaster_37_lm-5 mesial edge 37

arch_plaster_37_lm-6 distolingual dental cusp 37

arch_plaster_37_lm-7 mesiolingual dental cusp 37

arch_plaster_36_lm-1 distobuccal dental cusp 36

arch_plaster_36_lm-2 mesiobuccal dental cusp 36

arch_plaster_36_lm-3 distal edge 36

arch_plaster_36_lm-4 midpoint of central fissure 36

arch_plaster_36_lm-5 mesial edge 36

arch_plaster_36_lm-6 distolingual dental cusp 36

arch_plaster_36_lm-7 mesiolingual dental cusp 36

arch_plaster_35_lm-1 buccal dental cusp 35

arch_plaster_35_lm-2 distal edge 35

arch_plaster_35_lm-3 midpoint of central fissure 35

arch_plaster_35_lm-4 mesial edge 35

arch_plaster_35_lm-5 lingual dental cusp 35

arch_plaster_34_lm-1 buccal dental cusp 34

arch_plaster_34_lm-2 distal edge 34

arch_plaster_34_lm-3 midpoint of Zebtralfissure 34

arch_plaster_34_lm-4 mesial edge 34

arch_plaster_34_lm-5 lingual dental cusp 34

arch_plaster_33_lm-1 distal contact point 33

arch_plaster_33_lm-2 dental cusp 33

arch_plaster_33_lm-3 mesial contact point 33

arch_plaster_33_lm-4 tuberculum lingual 33

arch_plaster_32_lm-1 distal incisal edge 32

arch_plaster_32_lm-2 mesial incisal edge 32

arch_plaster_32_lm-3 tuberculum lingual 32

arch_plaster_31_lm-1 distal incisal edge 31

arch_plaster_31_lm-2 mesial incisal edge 31

arch_plaster_31_lm-3 tuberculum lingual 31

arch_plaster_41_lm-1 mesial incisal edge 41

arch_plaster_41_lm-2 distal incisal edge 41

arch_plaster_41_lm-3 tuberculum lingual 41

arch_plaster_42_lm-1 mesial incisal edge 42

arch_plaster_42_lm-2 distal incisal edge 42

arch_plaster_42_lm-3 tuberculum lingual 42

arch_plaster_43_lm-1 mesial contact point 43

arch_plaster_43_lm-2 dental cusp 43

arch_plaster_43_lm-3 distal contact point 43

arch_plaster_43_lm-4 tuberculum lingual 43

arch_plaster_44_lm-1 buccal dental cusp 44

arch_plaster_44_lm-2 mesial edge 44

arch_plaster_44_lm-3 midpoint of central fissure 44

arch_plaster_44_lm-4 distal edge 44

arch_plaster_44_lm-5 lingual dental cusp 44

arch_plaster_45_lm-1 buccal dental cusp 45

arch_plaster_45_lm-2 mesial edge 45

arch_plaster_45_lm-3 midpoint of central fissure 45

arch_plaster_45_lm-4 distal edge 45

arch_plaster_45_lm-5 lingual dental cusp 45

arch_plaster_46_lm-1 mesiobuccal dental cusp 46

arch_plaster_46_lm-2 distobuccal dental cusp 46

arch_plaster_46_lm-3 mesial edge 46

arch_plaster_46_lm-4 midpoint of central fissure 46

arch_plaster_46_lm-5 distal edge 46

arch_plaster_46_lm-6 mesiolingual dental cusp 46

arch_plaster_46_lm-7 distolingual dental cusp 46

arch_plaster_47_lm-1 mesiobuccal dental cusp 47

arch_plaster_47_lm-2 distobuccal dental cusp 47

arch_plaster_47_lm-3 mesial edge 47

arch_plaster_47_lm-4 midpoint of central fissure 47

arch_plaster_47_lm-5 distal edge 47

arch_plaster_47_lm-6 mesiolingual dental cusp 47

arch_plaster_47_lm-7 distolingual dental cusp 47

**Landmarks set along the dental root(s)**

In the following the landmarks for tooth 16 (FDI-scheme) is explained. All other teeth received landmarks according to this procedure.

tooth_16_lm-1 midpoint of toothroot at level of

Regression line

describes tooth

axis

the CEJL

tooth_16_lm-2 midpoint of toothroot 2 mm in direction

of apex

tooth_16_lm-3 midpoint of toothroot 4 mm in direction

of apex

tooth_16_lm-4 midpoint of toothroot 6 mm in direction

of apex

tooth_16_lm-5 apical end of root(s)
